# Predictors for blood loss in pediatric patients younger than 10 years old undergoing primary posterior hemivertebra resection: a retrospective study

**DOI:** 10.1186/s12891-019-2675-0

**Published:** 2019-06-22

**Authors:** Lulu Ma, Jianguo Zhang, Jianxiong Shen, Yu Zhao, Shugang Li, Xuerong Yu, Yuguang Huang

**Affiliations:** 1 Department of Anesthesiology, Peking Union Medical College Hospital, Beijing, 100730 China; 2 Department of Orthopedics, Peking Union Medical College Hospital, Beijing, 100730 China

**Keywords:** Congenital scoliosis, Hemivertebra resection, Blood loss

## Abstract

**Background:**

Blood loss during hemivertebra resection may be substantial. Few studies have examined the risk factors of blood loss undergoing hemivertebra resection, especially those in patients under 10 years old.

**Methods:**

Patients under 10 years old diagnosed with congenital scoliosis and hemivertebra were retrospectively included from January 2014 to October 2017. They all had primary posterior hemivertebra resection at Peking Union Medical College Hospital. Perioperative information was collected and multivariable linear logistic regression was performed to determine the independent risk factors of blood loss.

**Results:**

One hundred three patients were included. The mean total blood loss was 346 + 178 ml. The percentage of total blood loss to the EBV was 27.0 + 13.3%. Multivariable linear logistic regression indicated that preoperative total Cobb angle (*P* = 0.046) and the number of fused levels (*P* < 0.001) were independent risk factors of total blood loss. Preoperative platelet count and preoperative coagulation function were not associated with blood loss in patients undergoing hemivertebra resection.

**Conclusions:**

Preoperative total Cobb angle and the number of fused levels determined the blood loss for patients undergoing hemivertebra resection.

## Background

Hemivertebra is a common cause for congenital scoliosis. It has growth potential and can lead to spine deformity. Early detection and surgical intervention are recommended. Posterior resection of hemivertebrae with transpedicular instrumentation has been proven as a safe procedure for the correction of congenital scoliosis [1]. However, spine surgery is usually complex and associated with blood loss, placing patients at a high risk of allogeneic transfusion [2].

Allogenic transfusions have potential risks, including transfusion transmitted infection, fever, transfusion associated circulatory overload, immunologic and allergic reactions. Transfusions are also associated with increased postoperative complications, length of hospital stay and 30-day readmission rate for patients undergoing elective spine surgery [3].

Several strategies have been used to reduce the need for transfusion in spine surgeries: normovolemic hemodilution, controlled hypotension and antifibrinolytics. However, these techniques do have risks and they can’t eliminate the overall requirement for transfusion in spine surgeries. So it is necessary to understand which patients are at risk of massive blood loss for major spine surgery. Preoperative prediction of blood loss can help us make better preparation for potential bleeding, avoid unnecessary preoperative cross-match and waste of blood products.

However, there are few articles about blood management in patients with congenital scoliosis due to hemivertebrae, especially those under 10 years old. The aim of this study was to investigate the possible independent predictors of blood loss.

## Methods

One hundred three consecutive patients diagnosed with congenital scoliosis and hemivertebra were included in the study. They had primary posterior hemivertebra resection and infusion at Peking Union Medical College Hospital from January 2014 to October 2017. Exclusion criteria included prior spine operation, patients who had two hemivertebra resected and known bleeding problems.

Medical records of these patients were reviewed. All patients were induced with propofol (3 mg/kg), fentanyl (2μg/kg) and rocuronium (0.6 mg/kg). General anesthesia was maintained with continuous infusion of propofol and remifentanil. Fentanyl boluses (1-2μg/kg) were given when necessary for intraoperative analgesia. Blood pressure was maintained without decreasing lower than 20% of the preanesthetic value measured before induction of anesthesia. Ringers Lactate was used for fluid maintenance and colloid or blood products were administered when necessary. Tranexamic acid was not used. Intraoperative blood salvage with the bowl set of 70 ml (Haemonetics Cell Saver 5+, Haemonetics Corporation, Braintree, MA, USA) was used depending on the estimation of significant bleeding by surgeons.

All patients had posterior hemivertebra resection and bilateral transpedicular instrumentation in prone position. Intraoperative transcranial motor evoked potential (MEP) were used. For lumbar hemivertebra, one upper and one lower vertebra were fused. For thoracic hemivertebra, two upper and two lower vertebra were fused. In some instances, fused levels were extended to three or more upper and/or lower vertebra in case of severe scoliotic or kyphotic deformity. The posterior elements of hemivertebra, including the lamina, upper and lower facets and transverse process were removed. The rib head and the proximal part of the surplus rib on the convex side were also removed in patients with thoracic hemivertebra. Before the resection of vertebra body, a temporary rod was connected to screws on the concave side. The upper and lower discs, including the cartilage endplate were removed completely. The contra-lateral cartilage tissue of the hemivertebra was also resected. For patients with contralateral bar and rib synostosis, resection of bar and the synostosed rib heads was necessary. For patients with large hemivertebra or obvious kyphosis, a mesh cage filled autologous bone graft was needed to provide postoperative stability. Before closing, the drainage tube was placed underneath paravertebral muscles and disposable drainage bag was emptied every 24 h after the measurement of drainage volume**.** After operation, all patients were weaned from ventilator and extubated with the resume of adequate spontaneous ventilation and airway reflexes, minimal secretions and hemodynamic stability.

**Fig. 1:**
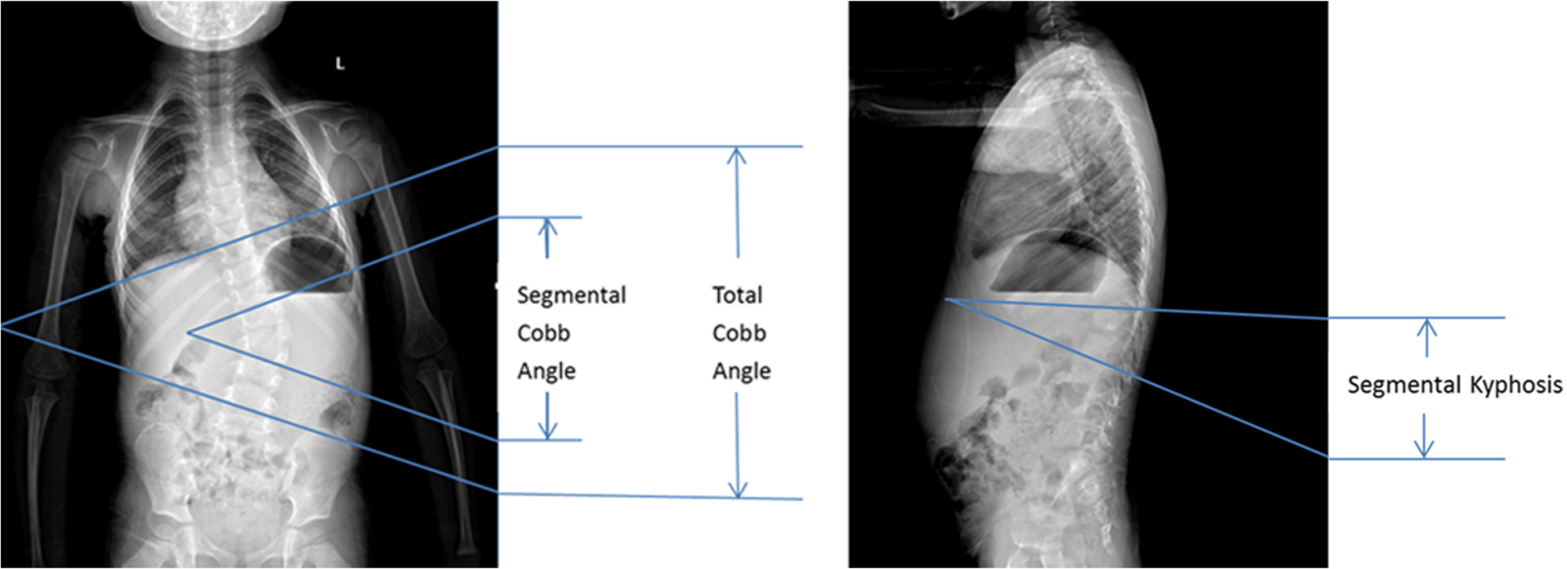
Angles measured in the anterior-posterior and lateral view: Segmental Cobb angle, total Cobb angle and segmental kyphosis

Patients’ information including age, sex, height, weight, past medical history, preoperative and postoperative segmental and total Cobb angles, segmental kyphosis or lordosis, fusion levels, operation time, estimated blood loss, intraoperative infusion of fluid, postoperative wound drainage and complications were collected. Cobb angles were measured according to the description by Ruf [1]. Segmental Cobb angle was measured from the upper endplate of the vertebra above the hemivertebra to the lower endplate of the vertebra below the hemivertebra. Total Cobb angle was measured from the upper endplate of the most tilted vertebra on top of the curve and the lower endplate of the most tilted vertebra on the bottom of the cure. Segmental kyphosis/lordosis was measured from the upper endplate above the hemivertebra to the lower endplate below the hemivertebra on the coronal plane (Fig. 1).

Laboratory tests which included blood cell count and coagulant function were also recorded. Total blood loss was calculated as the sum of intraoperative and postoperative blood loss. Intraoperative blood loss included the amount of blood in suction container and soaked sponge. And postoperative blood loss was estimated as the volume of wound drainage within 48 h after operation. The EBV was calculated as followings: body weight (kg) *75 ml/kg for patients ≤6 years old and body weight (kg) *70 ml/kg for patients > 6 years old [4, 5]. The percentage of total blood loss to EBV was used for the evaluation of blood loss.

Standardized statistical software (SPSS19, CHICAGO, IL) was used for statistical analysis. Data were reported as mean + standard deviation (SD) for normally distributed variables or median [25th, 75th interquartile range] for others. Potential predictor variables were first evaluated by calculating Pearson correlation coefficients, and multivariable linear logistic regression was performed to determine the independent risk factors of blood loss. *P* < 0.05 was defined as significant.

## Results

**Fig. 2:**
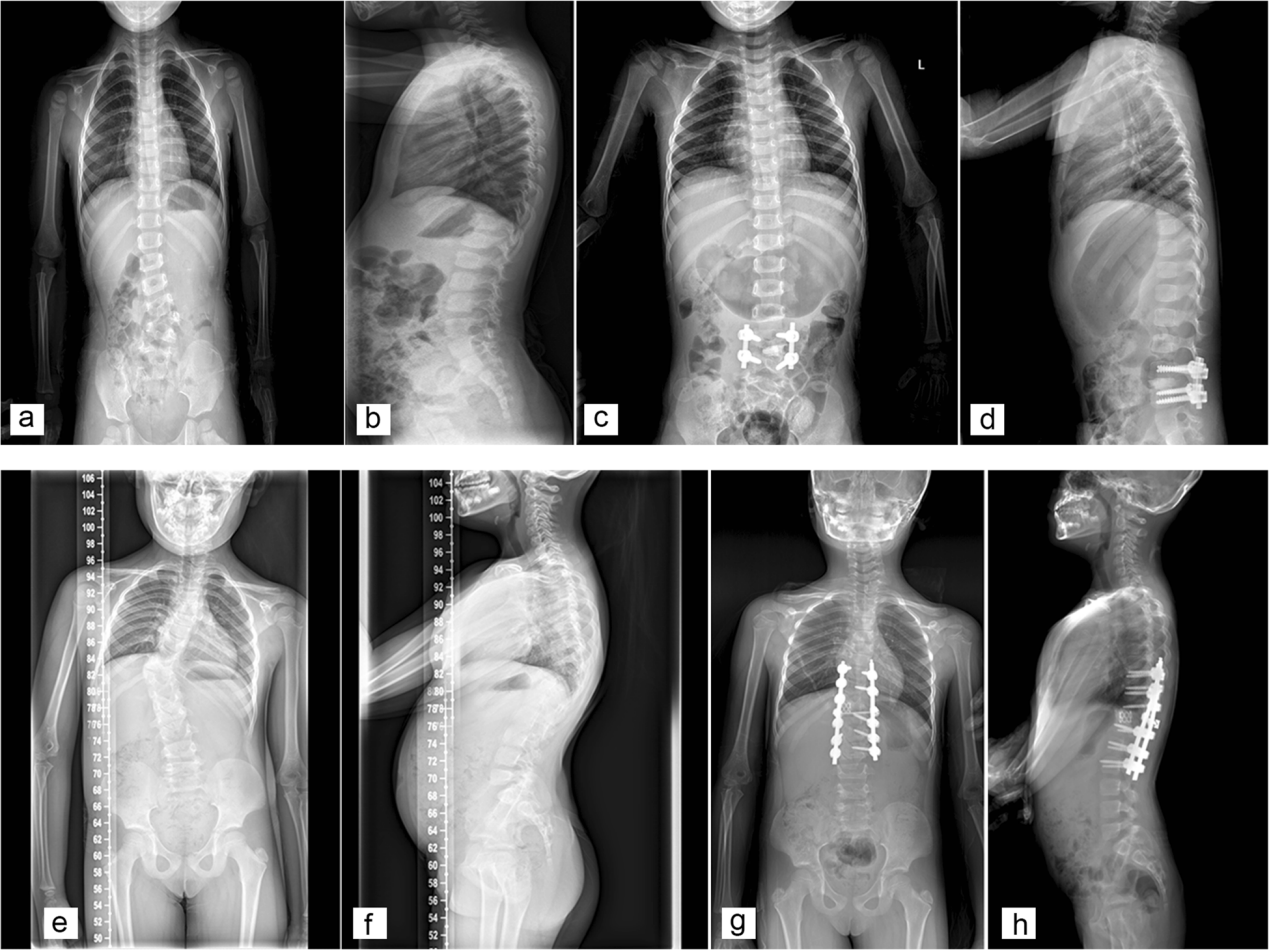
Preoperative and postoperative radiographs of two patients. **a**, **b**, **c**, **d**:a 2-year-old girl with a fully segmented hemivertebra at L4/5. **e**, **f**, **g**, **h**:a 7-year-old girl with a hemivertebra at T11

One hundred three consecutive patients, consisting of 45 girls and 58 boys were included in the study. The demographic characteristics and preoperative data were summarized in Table 1. Median age was 3 [2, 6] years old. The mean preoperative segmental and total Cobb angle and segmental kyphosis/lordosis were 40° + 12^°^, 35° + 12° and 20° + 15° respectively. Figure 2 showed preoperative and postoperative radiographs of two patients. .

**Table 1:** Demographic characteristics and preoperative data of patients (*n* = 103). Data were presented as mean + SD, median [25th, 75th interquartile range] or number (percentage)

Variables ^a^	
Sex	
Female	45(43.7%)
Male	58(56.3%)
Age(yr)	3 [2,6]
Height(cm)	96 [90,115]
Weight(Kg)	15.0 [13,22]
BMI	16.6 + 2.1
Segmental Cobb angle(°)	40 + 12
Total Cobb angle(°)	35 + 12
Segmental kyphosis(°)	20 + 15
Preoperative Hemoglobin(g/l)	130 + 11
Preoperative platelet count(^a^109)	305 + 66
Preoperative PT(s)	12.0 + 0.7
Preoperative APTT(s)	32.6 + 3.8
Preoperative fibrinogen(g/l)	2.3 + 0.5

^a^Mean + SD for normal distribution; median [25th,75th interquartile range] for others

The intraoperative and postoperative data were summarized in Table 2. The correction of segmental and total Cobb angle and segmental kyphosis/lordosis were 72.6% + 21.7%, 65.6 + 21.9 and 45.2 + 53.5% respectively. The mean average operation time was 176 + 40 min. The mean intraoperative blood loss, postoperative drainage volume and total blood loss were 225 + 142 ml, 121 + 78 ml and 346 + 178 ml respectively. The percentage of total blood loss to the EBV was 27.0 + 13.3%. 41(39.8%) patients used cell saver intraoperatively, and the overall allogenic transfusion rate was 73.8%.

**Table 2:** Intra-operative and post-operative data of patients (*n* = 103)

Variables ^*^	
Correction of segmental Cobb angle(%)	72.6 + 21.7
Correction of total Cobb angle(%)	65.6 + 21.9
Correction of segmental kyphosis/lordosis(%)	45.2 + 53.5
The number of fused levels	3[2,5]
Resection of ribs (n(%))	11(10.7%)
Length of surgery(minute)	176 + 40
Intraoperative blood loss(ml)	225 + 142
Postoperative drainage(ml)	121 + 78
Total blood loss(ml)	346 + 178
percentage of total blood loss to the EBV	27.0 + 13.3
Intraoperative cell saver use (n/%)	41(39.8%)
Intraoperative crystalloid (ml)	500[300,700]
Intraoperative colloid(ml)	0[0,100]
Allogenic transfusion(n(%))	76(73.8%)

Four cases of delayed wound healing and one case of numbness of lower extremities occurred. One patient had revision surgery due to displacement of internal fixation. There was no mortality and no complication related to blood loss or allogenic transfusion.

Multivariable linear logistic regression indicated that total Cobb angle (*P* = 0.046) and the number of fused levels (*P* < 0.001) were independent risk factors of total blood loss. (See Table 3).

**Table 3:** Univariable and multivariable linear regression models for blood loss

Predictor variable	Univariable		Multivariable	
	Pearson	*P* value	B(95% CI)	*P* value
Sex	0.026	0.793		
Age	−0.043	0.663		
Height(cm)	−0.079	0.425		
Weight(Kg)	−0.133	0.182		
BMI	− 0.146	0.141		
Segmental Cobb angle(°)	0.373	< 0.001*	0.001(−0.002–0.003)	0.596
Total Cobb angle(°)	0.474	< 0.001*	0.003(0.000–0.005]	0.046*
Segmental kyphosis(°)	0.085	0.396		
Correction of segmental Cobb angle(%)	0.022	0.829		
Correction of total Cobb angle(%)	−0.081	0.417		
Correction of segmental kyphosis/lordosis(%)	−0.084	0.400		
The number of fused levels	0.535	< 0.001*	0.036 (0.0147–0.054)	< 0.001*
Resection of ribs (n(%))	0.140	0.159		
Length of surgery(minute)	0.333	0.001*	−1.262E-5 (−0.001–0.001)	0.970
Preoperative Hemoglobin(g/l)	0.010	0.921		
Preoperative platelet count(*109)	−0.049	0.623		
Preoperative PT(s)	0.067	0.503		
Preoperative APTT(s)	−0.053	0.594		
Preoperative fibrinogen(g/l)	−0.057	0.570		

(*P < 0.05)

## Discussion

Blood loss with hemivertebrae resection is variable due to intraoperative exposure of paraspinal muscles, vertebral bodies and associated venous plexus. For pediatric patients younger than 10 years old, blood loss is very important because of lower weight, smaller blood volume and more sensitive to fluid loss or overdose when compared to adolescent or adult patients. There have been few reports on blood management in pediatric patients undergoing posterior resection of hemivertebra, especially in patients under 10 years old. Our study demonstrated that for patients undergoing primary posterior hemivertebra resection, perioperative blood loss was predicted by preoperative total Cobb angel and the number of fused levels.

In our study, blood loss was assessed by both intraoperative blood loss and postoperative drainage, while most previous studies focused on only intraoperative blood loss [6–8]. However bleeding usually lasts until the postoperative period for spine deformity surgeries [9]. And in our study, postoperative drainage counted for 35.6% (data not listed) of total blood loss, which also suggested the necessity of including postoperative drainage when assessing total blood loss. Both hemoglobin [10] and the need for blood transfusion [11] had been used to evaluate perioperative bleeding in hemivertebra resection. However, intravenous fluid intake can influence the value of hemoglobin and the criteria of transfusion at different hospitals and countries are variable, making them unreliable. Instead of the absolute value of total blood loss [12], we used the percentage of total blood loss to EBV for the evaluation of blood loss, which was more accurate by taking patients’ size into account. It is more reasonable to use the percentage when comparing blood loss of different studies and it is more valuable in determining transfusion. The ratio of total blood loss to EBV was 27.0% in our study, which was in accordance with previous report [12].

Previous studies have identified factors associated with increased blood loss in scoliosis, which include larger Cobb angles [9, 13], the number of segments fused [14] and osteotomy [13]. For patients undergoing one hemivertebra resection, the independent risk factors for blood loss are the preoperative total Cobb angel and the number of fused levels. Both reflect the severity of scoliosis. And the more levels are fused, the more muscle and vertebra are exposed.

Transfusion rate was 73.8% in our case series. The reasons of high transfusion rate were as the followings: First, all patients had osteomy. Osteomy had been confirmed as the risk factor of blood loss and transfusion in adolescent idiopathic scoliosis patients [13]. Secondly, intraoperative monitoring is essential for spine surgery, and transcranial motor evoked potential (MEP) was used in our series to prevent spinal cord injury. However, due to the immaturity of motor nervous pathways in young children, the successful rate of baseline of MEP was lower, and the waveform and amplitude were poor when compared to those in adolescent patients [15]. Hypotension and anemia due to hemorrhage may also lead to MEP deterioration. Expanding intravascular volume and restoring hemoglobin level (9-10 g/dl) are necessary [16]. Tranexamic acid was not used in our case series. Although tranexamic acid has been confirmed to decrease blood loss in adolescent idiopathic scoliosis [17] and pediatric vertebral column resection [18], its safety and dosage guidelines among children, especially those under 10 years old are unknown.

Preoperative platelet count and coagulation function were not related to blood loss in our study. Decreased platelet quantity was detected in patients during spine surgeries [19], however thrombocytopenia had limited effect on bleeding in patients with idiopathic scoliosis undergoing spine deformity surgeries [20]. Hypercoagulable state followed by fibrinolysis [21] was reported in patients after spinal surgeries. Besides these, preoperative fibrinogen concentration was proven to be associated with perioperative bleeding and transfusion [11]. We didn’t find the relationship between preoperative fibrinogen level and bleeding in our study. And the role of coagulation mechanism in the bleeding of hemivertebra resection remains unclear.

There are several limitations in our study. First, this is a retrospective study with limited number of patients. Second, it was a single-center study and the validity of our results was limited.

## Conclusions

In conclusion, preoperative total Cobb angle and the number of fused levels are the predictor for blood loss for patients undergoing one hemivertebra resection surgery. Further researches are necessary to determine the role of blood management in decreasing blood loss and transfusion in patients undergoing hemivertebra resection.

## Data Availability

The datasets used and/or analysed during the current study are available from the corresponding author on reasonable request.

## Abbreviations

APTT: Activate partial thromboplastin time
EBV: Estimated blood volume
INR: International normalized ration.
MEP: Motor evoked potential
PT: Prothrombin time
SD: Standard deviation
